# Association Between Postoperative Pain Intensity and Delirium in Cardiac and Neurosurgical Patients: A Retrospective Pilot Study

**DOI:** 10.3390/jcm14248840

**Published:** 2025-12-13

**Authors:** Mateusz Szczupak, Jacek Kobak, Jakub Wiśniewski, Jolanta Wierzchowska, Sabina Krupa-Nurcek

**Affiliations:** 1 Department of Anesthesiology and Intensive Care, Copernicus Hospital in Gdańsk, 80-803 Gdańsk, Poland; szczupak.mateusz@gmail.com (M.S.); jwierzchowska@wss.gda.pl (J.W.); 2 Department of Otolaryngology, Faculty of Medicine, Medical University of Gdańsk, Mariana Smoluchowskiego 17 Street, 80-214 Gdańsk, Poland; 3 Department of Neurosurgery, Copernicus Hospital in Gdańsk, 80-803 Gdańsk, Poland; drjakubwisniewski@gmail.com; 4 Department of Surgery, Faculty of Medicine, Collegium Medicum, University of Rzeszów, 35-959 Rzeszów, Poland; sabinakrupa@o2.pl

**Keywords:** postoperative pain, delirium, cardiac surgery, neurosurgery, intensive care, risk factors

## Abstract

**Background/Objective**: Postoperative pain and delirium are frequent and clinically relevant complications in patients undergoing major cardiac or neurosurgical procedures. The interaction between these conditions remains insufficiently characterized, particularly across heterogeneous surgical populations. This study aimed to investigate the relationship between postoperative pain intensity and delirium severity within the first 48 h after surgery in cardiac and neurosurgical patients. **Methods**: This retrospective observational analysis included 408 individuals—202 following cardiac surgery and 206 after neurosurgical procedures. Pain intensity was measured using the Numerical Rating Scale (NRS), while delirium presence and severity were assessed using the CAM-ICU and CAM-ICU-7 instruments. Associations between NRS scores, delirium severity, demographic characteristics, and ICU length of stay were examined. **Results**: Cardiac surgery patients experienced higher pain levels on postoperative day 1 compared with neurosurgical patients; this difference was not observed on day 2. In the cardiac cohort, higher NRS scores were positively associated with greater delirium severity on both postoperative days. No such association was detected in the neurosurgical group. Pain scores also differed across procedure types within each specialty, and several demographic variables (age, sex, ICU stay duration) were linked with variations in pain intensity. On postoperative day 1, pain intensity showed a moderate association with delirium severity (Spearman ρ = 0.23; 95% CI 0.14–0.32). Patients who developed delirium had higher pain scores (r = 0.25). In ordinal logistic regression, greater pain on postoperative day 1 independently predicted higher delirium severity (OR 2.24; 95% CI 1.70–2.94). **Conclusions**: Significant associations between postoperative pain intensity and delirium severity were identified in cardiac surgery patients, whereas no similar pattern emerged among neurosurgical patients. Given the retrospective design and incomplete data on perioperative pharmacotherapy, the findings should be interpreted descriptively and do not support causal conclusions. These results underscore the importance of systematic monitoring of pain and cognitive function in high-risk postoperative populations and highlight the need for prospective studies to elucidate the complex interplay between pain, perioperative factors, and postoperative delirium.

## 1. Introduction

Postoperative delirium is widely recognized as one of the most consequential and difficult-to-manage complications following procedures performed under general anesthesia [1,2]. It is classified as a cognitive disturbance characterized by an abrupt onset of fluctuating deficits in attention and consciousness [3]. Symptoms typically appear rapidly—ranging from approximately 10 min after emergence from anesthesia to as long as 7 days after surgery—and often follow a variable day-to-day trajectory [4].

In the general surgical population, postoperative delirium occurs in an estimated 2.5–3% of patients [5,6]. Its prevalence increases substantially among older adults, particularly those aged 60–70 years, in whom rates reach 10–20% [7,8,9]. This condition carries meaningful clinical consequences: it prolongs total hospitalization by an additional 2–3 days and extends intensive care unit stays by roughly 2 days on average [10,11,12].

The relationship between postoperative pain and delirium is multifaceted, bidirectional, and shaped by biological, psychological, and behavioral factors, including the effects of depression and disturbed sleep [13]. Although multiple perioperative contributors can precipitate delirium, acute cerebral stress is considered a key underlying mechanism [14]. Chronic pain is known to alter cortisol regulation and sustain elevated concentrations of pro-inflammatory cytokines. Evidence indicates that both acute and chronic pain reduce the threshold for developing delirium, thereby heightening patient susceptibility [15].

Postoperative delirium is a multifactorial neuropsychiatric syndrome arising from the interplay of patient-specific vulnerability factors and perioperative precipitating insults. Contemporary models emphasize that delirium reflects an acute failure of cerebral homeostasis driven by neuroinflammation, impaired neuronal network connectivity, blood–brain barrier dysfunction, cholinergic deficiency, oxidative stress, metabolic derangements, altered sleep–wake regulation, and disturbances in melatonin secretion [3,14]. Large systematic reviews demonstrate that postoperative delirium results from a convergence of multiple predisposing factors—advanced age, frailty, pre-existing cognitive decline, sensory impairment, multimorbidity, and polypharmacy—and precipitating factors such as depth and duration of anesthesia, hemodynamic instability, hypoxia, metabolic abnormalities, infection, sedative and analgesic exposure, surgical invasiveness, and neuroinflammatory stress [16,17]. Importantly, the perioperative pain response is itself a potent neurobiological stressor: acute pain activates the hypothalamic–pituitary–adrenal axis, increases circulating cytokines (IL-6, TNF-α), amplifies microglial activation, and has been shown to lower the delirium threshold in susceptible individuals [13].

Given this complexity, postoperative pain represents only one of many interconnected mechanisms contributing to delirium; however, it remains a clinically relevant, potentially modifiable precipitant, especially in surgical cohorts characterized by significant inflammatory burden and physiological stress, such as cardiac and neurosurgical patients. Prior research suggests that inadequate pain control, heightened pain variability, and dysregulated nociceptive signaling may exacerbate attentional deficits and precipitate acute cognitive dysfunction [18]. Despite this, few studies directly compare the pain–delirium interplay across distinct high-risk surgical specialties. Therefore, we focused specifically on postoperative pain as an initial exploratory factor within a broader delirium framework to better understand how early postoperative nociceptive burden may relate to delirium severity in cardiac and neurosurgical patients.

Surgical innovations over recent decades have significantly broadened the scope of interventions performed, particularly in cardiac and neurosurgical practice [19]. The present study focuses on patients undergoing the most frequently performed procedures in these specialties. In cardiac surgery, these included aortic valve replacement (AVR), coronary artery bypass grafting (CABG), the Bentall procedure—entailing replacement of the aortic valve, ascending aorta, and aortic root with reimplantation of the coronary ostia—and minimally invasive mitral valve plasty (miniMVpl) performed through a small right thoracic approach.

In neurosurgery, representative procedures comprised management of discopathy (degenerative disc disease), brain tumor resections, microdiscectomy as a minimally invasive spinal technique, thermolesion for percutaneous thermal pain modulation, surgery for spinal cord injuries, vertebroplasty involving cement augmentation of a compromised vertebra, and interventions for hydrocephalus [20].

Postoperative delirium, positioned at the convergence of increasingly complex surgical techniques and multifactorial perioperative risks, continues to represent a major challenge in perioperative care. Improving its early detection, understanding, and prevention remains essential for optimizing clinical outcomes and enhancing the efficiency of healthcare systems.

## 2. Methods

### 2.1. Participants

The analysis included 408 individuals: 202 patients who underwent cardiac surgery and 206 who underwent neurosurgical procedures. The cardiac cohort consisted of patients treated for various structural heart conditions, including aortic valve replacement (AVR), the Bentall procedure, coronary artery bypass grafting (CABG), and minimally invasive mitral valve repair (miniMVpl). The neurosurgical group included patients managed for discopathy, brain tumors, lumbar microdiscectomy, thermolesion procedures, spinal cord injuries, vertebroplasty, and hydrocephalus.

### 2.2. Procedures

The data collection period extended from early April 2024 through the end of August 2024. During this time, the research team reviewed medical documentation to identify episodes of pain and delirium within the study population. Before initiating the analysis, approvals were obtained from the Directors of the participating institutions, followed by a favorable opinion from the Bioethics Committee. Once these authorizations were secured, the evaluation of records from cardiac and neurosurgical patients commenced. The review encompassed medical files, laboratory results, and nursing notes. Any patient records lacking essential information—such as documented pain scores or delirium assessments—were excluded from the final analysis.

### 2.3. Statistical Analysis

All statistical procedures were performed in accordance with current methodological recommendations for studies involving ordinal outcomes and repeated postoperative measurements. Descriptive statistics were reported as means with standard deviations or medians with interquartile ranges, depending on data distribution.

Pain intensity was assessed using the Numerical Rating Scale (NRS). Delirium severity was evaluated with the CAM-ICU-7 scale and categorized into three levels: no delirium (0–2 points), mild/moderate delirium (3–5 points), and severe delirium (6–7 points). Delirium presence was defined using the CAM-ICU algorithm (positive CAM-ICU screening). The CAM-ICU-7 scale was used exclusively to quantify delirium severity.

Because NRS values and CAM-ICU-7 scores are ordinal or non-normally distributed, non-parametric methods were used. Associations between pain intensity and delirium severity were examined using Spearman rank correlations (with 95% confidence intervals). Group comparisons were performed using the Mann–Whitney U test (reported with rank-biserial effect size *r*) and the Kruskal–Wallis test. When the Kruskal–Wallis test indicated significant differences, Dunn’s post hoc tests with Holm correction for multiple comparisons were applied.

To evaluate independent predictors of postoperative delirium, a multivariable logistic regression model was constructed with delirium as the dependent variable. Covariates included age, sex, procedure type (cardiac vs. neurosurgical), number of comorbidities, and ICU length of stay (hours). Results were presented as odds ratios (OR) with 95% confidence intervals.

To assess the association between pain and delirium severity, a proportional odds (ordinal logistic) regression model was applied using the three-level CAM-ICU-7 scale as the outcome, with NRS on postoperative day 1, age, sex, and procedure type as predictors.

Because pain intensity was measured repeatedly at seven time points on postoperative day 1, linear mixed-effects models were used to account for within-patient correlation. The model included random intercepts for patients and fixed effects for time and delirium presence. A total of 2856 NRS observations (408 patients × 7 time points) were included in the mixed-effects analysis.

All tests were two-tailed. Statistical significance was defined as *p* < 0.05, with multiple comparisons adjusted using the Holm method. Analyses were performed using IBM SPSS Statistics 23.0 for basic procedures and Python 3.14.2 (statsmodels, scipy, scikit-posthocs) for ordinal regression, mixed-effects modeling, and Dunn’s post hoc tests.

### 2.4. Ethics

Ethical approval for this study was granted by the Research Ethics Committee of the Regional Medical Chamber in Gdańsk (protocol no. KB-6/24). All participants were fully informed about the study’s aims and provided written consent prior to enrolment.

The authors confirm that the study procedures complied with all relevant institutional regulations, national legislation, and ethical standards.

### 2.5. Research Team and Reflexivity

Five researchers conducted the study. After receiving the necessary consents, the investigators divided into two groups (two people in the neurosurgery department and 2 in the cardiac surgery department) and analyzed the documentation, which included information on the patients’ pain levels and delirium episodes.

### 2.6. Study Population and Eligibility Criteria

Initially, 468 patients were evaluated for eligibility. Sixty individuals were removed from the analysis because of insufficient medical records, pre-existing neurological or psychiatric conditions, or absence of postoperative NRS assessments. The final cohort included 408 participants—202 who underwent cardiac surgery and 206 who underwent neurosurgical procedures.

Inclusion criteria

- Age ≥ 18 years;
- Elective or urgent cardiac or neurosurgical procedure under general anesthesia;
- Availability of perioperative pain measurements using the NRS;
- Postoperative delirium assessment using the CAM-ICU and CAM-ICU-7 scales.

Exclusion criteria

- Age < 18 years;
- Pre-existing dementia, psychiatric disorders, or alcohol/substance abuse;
- Active infection or fever on admission;
- Chronic inflammatory or autoimmune diseases;
- Active malignancy;
- No pain intensity data on the NRS or CAM-ICU;
- Use of psychotropic medications, benzodiazepines, and anticholinergics.

**Figure 1:**
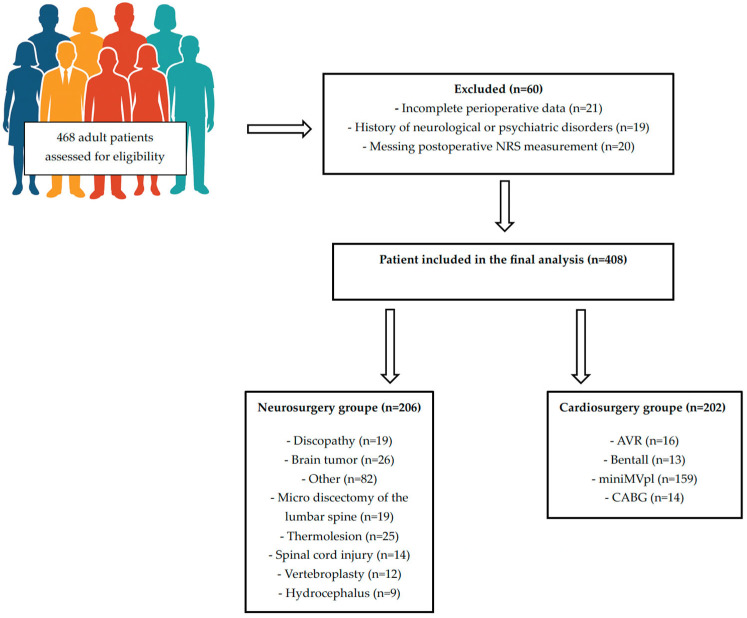
Flowchart study.

The patient selection process and final allocation are summarized in Figure 1 (Study Flowchart).

### 2.7. Rationale for Selecting Cardiac and Neurosurgical Procedures

Cardiac and neurosurgical procedures were chosen because they are among the surgical specialties with the highest rates of postoperative delirium and intense postoperative pain, both of which are influenced by systemic and neuroinflammatory pathways. In cardiac surgery, especially when cardiopulmonary bypass is used, patients are exposed to a marked systemic inflammatory reaction, transient episodes of cerebral hypoperfusion, and microembolic phenomena. These mechanisms are strongly linked to the development of delirium and may also exacerbate postoperative pain.

Neurosurgical procedures, on the other hand, inherently involve manipulation of central nervous system structures, which can trigger neuroinflammatory responses, local tissue irritation, disturbances in circadian rhythm regulation, and significant postoperative discomfort.

Examining these two distinct surgical cohorts—each characterized by clinically relevant inflammatory and neurological stressors—allowed for an assessment of how postoperative pain, inflammatory activity, and delirium interact in different yet pathophysiologically comparable contexts.

### 2.8. Data Collection and Variables

Data were obtained from medical and nursing records, databases, and perioperative records. The variables collected included:

- Demographic data: age, gender;
- Clinical data: type and duration of surgery, length of stay in the intensive care unit, and total hospital stay;
- Pain assessment: Numerical Rating Scale (NRS);
- Delirium assessment: Confusion Rating Method for the ICU (CAM-ICU);
- Delirium severity: Confusion Rating Method—Intensive Care Unit—7 (CAM-ICU-7);
- Analgesic medications used.

### 2.9. Definitions of Key Variables

Postoperative delirium was defined as a sudden, fluctuating disturbance in attention and cognitive function, assessed using the CAM-ICU protocol. Evaluations were conducted twice daily (at 08:00 and 20:00) by ICU nurses who had completed formal training in the instrument, with each screening subsequently reviewed and confirmed by an anesthesiologist. The CAM-ICU algorithm assesses four core domains:

1. Acute onset or fluctuating course—identification of a new change in mental status within the past 24 h, or verification that previously noted abnormalities exhibited a waxing and waning pattern.
2. Inattention—examined through structured concentration tasks, such as the SAVEAHAART letter-recognition test, or by observing non-verbal command responses.
3. Disorganized thinking—evaluated with short logical questions and simple task commands (e.g., “Does a stone float on water?” or “Count from one to five”).
4. Altered level of consciousness—determined using the Richmond Agitation–Sedation Scale (RASS); any value other than 0 (“calm and alert”) indicates impaired arousal.

Delirium was diagnosed when the patient fulfilled both the acute onset/fluctuation criterion and the inattention criterion, along with either disorganized thinking or an altered level of consciousness, consistent with the CAM-ICU diagnostic structure.

Delirium severity was measured using the CAM-ICU-7 scale, which converts the CAM-ICU domain ratings into a composite score ranging from 0 to 7. Scores of 0–2 reflect no delirium, values of 3–5 indicate mild to moderate delirium, and scores of 6–7 correspond to severe delirium [21].

Pain intensity was assessed using the 11-point Numeric Rating Scale (NRS), where 0 signifies no pain and 10 represents the worst pain imaginable. Measurements were recorded every 8 h during the first 48 h after surgery, as well as whenever patients reported discomfort.

Postoperative day 1 (POD1) was defined as the first 24 h interval following ICU admission after surgery (0–24 h), whereas postoperative day 2 (POD2) covered the subsequent 24 h (24–48 h). All evaluations were assigned to POD1 or POD2 strictly according to the timestamp documented in the nursing records.

### 2.10. Perioperative Analgesia, Sedation, and Anesthesia Protocols

This study was based on retrospective medical record analysis, detailed quantitative data on perioperative and postoperative pharmacotherapy (opioid dosing in MME/day, benzodiazepine equivalents, anticholinergic burden, propofol/dexmedetomidine infusions, or antipsychotic exposure) were not consistently documented in all patient files. To enhance transparency and help contextualize potential confounding, the standard analgesia and sedation protocols used in both participating units during the study period are described below.

Cardiac surgery unit protocol:

- Postoperative analgesia followed a multimodal regimen consisting of oxycodone (intravenous or oral), transdermal buprenorphine, and acetaminophen administered at scheduled intervals. Metamizole was used as an adjunct where appropriate. Regional or neuraxial anesthesia techniques were not employed for cardiac surgery patients during the analyzed period. Short-term postoperative sedation in the ICU was achieved using propofol when clinically required. Benzodiazepines were not used for routine postoperative sedation. Antipsychotics were reserved exclusively for cases of severe agitation that posed safety risks.

Neurosurgical unit protocol:

- Pain management consisted of acetaminophen, metamizole, and rescue doses of oxycodone. Continuous sedative infusions were not routinely used following neurosurgical procedures. Dexmedetomidine, benzodiazepines, and anticholinergic agents were not part of the standard postoperative care pathway. Antipsychotic medications were administered only for clinically significant behavioral disturbance.

Because individual dosing, timing, and exposure duration were not reliably documented, these variables could not be included in statistical adjustment. This limitation has been explicitly discussed in the limitations section.

### 2.11. Handling of Missing Data and Assessment Completeness

In the analytic dataset, both delirium and pain assessments demonstrated exceptionally high completeness. Delirium was evaluated using the CAM-ICU and CAM-ICU-7 instruments. CAM-ICU-7 scores were categorized into three severity levels: 0–2 indicating no delirium, 3–5 indicating mild to moderate delirium, and 6–7 indicating severe delirium. These categories were available for all patients on both postoperative day 1 (POD1) and postoperative day 2 (POD2).

There were no missing values in the binary delirium indicators (“presence of delirium”) or in the three-level CAM-ICU-7 severity variables for either POD1 or POD2. On POD1, 346 patients (84.8%) had no delirium, 18 (4.4%) had mild to moderate delirium, and 44 (10.8%) had severe delirium. On POD2, 378 patients (92.6%) had no delirium, 19 (4.7%) had mild to moderate delirium, and 11 (2.7%) had severe delirium. Thus, all 408 patients contributed valid delirium data for both postoperative days, and no imputation was required.

According to the clinical protocol, CAM-ICU and CAM-ICU-7 assessments were scheduled twice daily (08:00 and 20:00). However, in the exported research dataset these assessments were stored as day-level severity categories rather than individual time-stamped entries. Although this prevented reconstruction of completed assessments at specific clock times, at least one valid CAM-ICU-7 assessment was available for every patient on both POD1 and POD2.

Pain intensity was assessed using the 11-point Numerical Rating Scale (NRS) at seven predefined time points per day (08:00, 12:00, 15:00, 18:00, 22:00, 02:00, and 06:00). This schedule yielded 2856 planned NRS measurements per day (408 patients × 7 time points). All scheduled NRS assessments were completed on both POD1 and POD2 (2856/2856 per day; 5712/5712 total), with no missing NRS values in any of the predefined time windows.

All analyses involving delirium and pain were therefore performed as complete-case analyses. The full availability of delirium and NRS data indicates that attrition is unlikely to have biased delirium estimates or the observed associations between pain and delirium. The lack of time-stamped CAM-ICU-7 measurements is acknowledged as a data-structural limitation inherent to the retrospective extraction process.

## 3. Results

A total of 408 patients were included in the analysis, comprising 202 individuals after cardiac surgery (49.51%) and 206 patients undergoing neurosurgical procedures (50.49%). All delirium assessments (CAM-ICU and CAM-ICU-7) and all NRS pain measurements scheduled across POD1 and POD2 were fully completed, with no missing values at any time point.

The study population consisted of 181 women and 227 men. Among cardiac patients, the ages ranged from 18 to 78 years (SD = 13.83), whereas neurosurgical patients were aged 18 to 71 years (SD = 11.82). Postoperative ward stays varied from 17 to 172 h in the cardiac cohort (SD = 24.26), with the same maximum duration of 172 h observed in the neurosurgical cohort (SD = 27.82). Within the cardiac surgery group, 16 patients (7.92%) underwent aortic valve replacement (AVR), 13 (6.44%) underwent the Bentall procedure, 159 (78.71%) received minimally invasive mitral valve plasty (miniMVpl), and 14 (6.93%) underwent coronary artery bypass grafting (CABG). In the neurosurgical cohort, 19 patients (9.22%) were treated for lumbar discopathy with microdiscectomy, 26 (12.62%) underwent resection of a brain tumor, thermolesion was performed in 25 patients (12.14%), spinal cord injury surgery in 14 (6.80%), vertebroplasty in 12 (5.83%), and 9 patients (4.37%) were operated on for hydrocephalus. The remaining 82 individuals (39.81%) underwent other neurosurgical procedures. An overview of the baseline characteristics and grouping variables is provided in Table 1.

### 3.1. Pain Level in Individual Days and Procedures

In the analyzed cohort, pain intensity scores ranged from 0 (no pain) to 10 (maximum imaginable pain). Among study participants, reported values fell within the interval of 1 to 8. On postoperative day 1, patients after cardiac surgery had a significantly higher mean pain score than neurosurgical patients (*p* < 0.00001). By postoperative day 2, this difference was no longer statistically significant (*p* = 0.43).

Within the cardiac surgery group, a marked reduction in pain intensity was observed between day 1 (5.32 ± 1.25) and day 2 (4.52 ± 1.14), reaching statistical significance (*p* < 0.00001). In contrast, neurosurgical patients showed no meaningful change in pain levels between the two time points (*p* = 0.47). A detailed summary of these findings is provided in Table 2.

#### 3.1.1. Cardiac Surgery Patients

Analysis of NRS pain scores revealed significant differences among cardiac surgery subgroups on both postoperative day 1 and day 2. Patients undergoing the miniMVpl procedure reported markedly lower pain intensity compared with all other cardiac procedures at both time points. Additionally, a significant reduction in mean pain scores from day 1 to day 2 was observed for patients after AVR, Bentall, and CABG procedures (Table 3).

#### 3.1.2. Neurosurgery Patients

In the neurosurgical cohort, significant differences in mean pain intensity across procedure types were observed on postoperative day 1. Furthermore, a statistically significant reduction in NRS pain scores from day 1 to day 2 was noted for patients undergoing brain tumor surgery, lumbar microdiscectomy, thermolesion, spinal cord injury procedures, as well as for those classified under other neurosurgical interventions (Table 3).

### 3.2. Occurrence of Delirium in Patient Groups and Treatment Groups on the First and Second Day

Delirium severity was evaluated using the CAM-ICU-7 scoring system, where values of 0–2 indicate absence of delirium, 3–5 reflect mild to moderate symptoms, and 6–7 correspond to severe delirium. In the analyzed cohort, delirium scores ranged from 0 (no delirium) to 7 (severe delirium). Mean CAM-ICU-7 values were significantly higher in the cardiac surgery group compared with the neurosurgical group on both postoperative day 1 and day 2 (Table 4).

The distribution of delirium severity on postoperative days 1 and 2 was compared between the cardiac and neurosurgical cohorts. Significant differences were observed on both days (*p* < 0.000001). Cardiac surgery patients showed a noticeably higher frequency of mild to moderate delirium, and severe delirium occurred more often in this group on both postoperative days (Table 4).

#### 3.2.1. Cardiac Surgery Patients

Among cardiac surgery patients, the distribution of delirium severity differed significantly across procedure types on postoperative day 1. The miniMVpl group demonstrated the highest proportion of severe delirium, reaching 7.43%. In comparison, severe delirium occurred in 3.96% of patients after AVR and in 3.96% after the Bentall procedure, while no severe delirium cases were identified following CABG. By postoperative day 2, differences in delirium occurrence among cardiac procedures were no longer statistically significant (*p* = 0.07).

#### 3.2.2. Neurosurgery Patients

In the neurosurgical cohort, delirium severity varied significantly across procedure types on postoperative day 1. The highest proportion of severe delirium was observed in patients undergoing spinal cord injury surgery (5.83%). In contrast, severe delirium occurred in fewer than 0.49% of patients after lumbar microdiscectomy. On postoperative day 2, no statistically significant differences in delirium incidence were detected among the neurosurgical procedures (*p* = 0.68) (Table 5).

A visual comparison of CAM-ICU-7 scores across cardiac and neurosurgical procedures revealed distinct patterns of distribution rather than consistent differences in central tendency. Cardiac procedures demonstrated predominantly low delirium severity, with narrow interquartile ranges and limited variability on both postoperative days.

Neurosurgical procedures exhibited greater heterogeneity, with wider score distributions and occasional higher CAM-ICU-7 values, particularly on POD1. This suggests that delirium severity in the neurosurgical cohort is more procedure-dependent and characterized by a broader range of postoperative responses.

**Figure 2:**
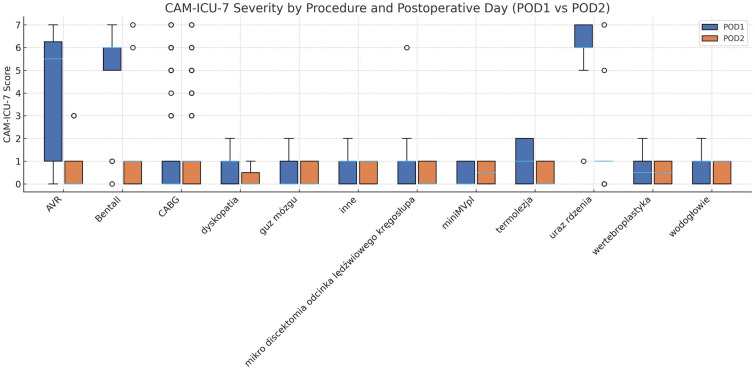
Distribution of CAM-ICU-7 delirium severity across cardiac and neurosurgical procedures on postoperative day 1 and postoperative day 2.

These distributional patterns are depicted in Figure 2 and complement the statistical analyses presented in the preceding section, without implying differences not supported by formal testing.

### 3.3. Relationship of NRS Pain Score Level with Patient Delirium Level

Associations between postoperative pain intensity and delirium severity were evaluated using non-parametric methods due to the ordinal distribution of both NRS and CAM-ICU-7 scores. On postoperative day 1, pain intensity was positively correlated with delirium severity, as demonstrated by a significant Spearman rank correlation (ρ = 0.23; 95% CI 0.14–0.32; *p* < 0.001 after Holm correction). No significant correlation was observed on postoperative day 2 (ρ = 0.04; *p* = 0.4546). Detailed correlation data are presented in Table 6.

When comparing pain levels between patients with and without delirium, individuals who developed delirium demonstrated significantly higher NRS scores on postoperative day 1 (median 6.00 vs. 5.29; Mann–Whitney U = 8442.5; r = 0.25; *p* < 0.001 after Holm correction). This difference was not significant on postoperative day 2 (*p* = 0.1747). The results of the Mann–Whitney U analysis are shown in Table 7.

The Kruskal–Wallis test further supported a monotonic increase in pain intensity across delirium severity categories. On postoperative day 1, NRS values differed significantly among the three CAM-ICU-7 groups (H = 39.57; η^2^ = 0.097; *p* < 0.001). A similar but less pronounced pattern was observed on postoperative day 2 (H = 11.90; η^2^ = 0.029; *p* = 0.0078). Full Kruskal–Wallis results for postoperative days 1 and 2 are presented in Table 8 and Table 9.

Dunn’s post hoc tests identified which severity categories differed significantly. On postoperative day 1, both mild/moderate and severe delirium groups had significantly higher pain levels compared with the no-delirium group, whereas no difference was observed between the two delirium subgroups. On postoperative day 2, only the severe delirium group differed significantly from the no-delirium group. Detailed pairwise comparisons for postoperative days 1 and 2 are provided in Table 10 and Table 11, respectively.

Finally, a linear mixed-effects model incorporating all seven NRS assessments from postoperative day 1 (2856 observations from 408 patients) demonstrated that delirium presence remained an independent predictor of higher pain intensity throughout the entire 24 h period (β = 1.23; 95% CI 0.99–1.48; *p* < 0.001), even after controlling for time effects and patient-level variability. These results confirm the consistency of the association across repeated measures (Table 12).

### 3.4. Multivariable Logistic Regression for Delirium Presence

In multivariate analysis, the type of procedure was the only independent predictor of delirium. Compared with cardiac surgery patients, neurosurgery patients had a significantly lower adjusted risk of delirium (OR 0.15; 95% CI 0.08–0.29; *p* < 0.001). Age, gender, number of comorbidities, and duration of ICU stay were not significant predictors. Detailed data are presented in Table 13.

### 3.5. Ordinal Logistic Regression for CAM-ICU-7 Delirium Severity

According to the ordinary model (Table 2), greater pain on postoperative day 1 was independently associated with greater delirium severity (OR 2.24; 95% CI 1.70–2.94; *p* < 0.001). Detailed data are presented in Table 14.

### 3.6. Stratification of Major Procedure Families

In response to the heterogeneity of surgical procedures in both cohorts, an additional stratified analysis was performed by grouping operations into clinically coherent families with comparable surgical invasiveness, pain trajectories, and delirium risk.

#### 3.6.1. Cardiac Surgery Cohort: Sternotomy vs. Minimally Invasive Mini-Thoracotomy

Cardiac procedures were classified into two major groups:

- Sternotomy procedures: AVR, Bentall, CABG.
- Minimally invasive mini-thoracotomy: miniMVpl.

This stratification reflects major differences in surgical trauma, postoperative pain intensity, and systemic inflammatory response.

Pain intensity

- On postoperative day 1, sternotomy procedures demonstrated higher mean NRS values compared with miniMVpl (data consistent with Table 3).
- On postoperative day 2, the difference narrowed but remained clinically noticeable.

Delirium occurrence

- The sternotomy group showed a higher proportion of both mild–moderate and severe delirium on day 1.
- MiniMVpl procedures, despite lower pain scores, paradoxically exhibited several cases of severe delirium, consistent with Table 5.
- On day 2, differences in delirium distribution between groups diminished.

Interpretation

These findings demonstrate that procedure invasiveness influences both postoperative pain and delirium but does not fully explain the variability in delirium occurrence, suggesting contributions from age, inflammatory response, and individual vulnerability.

#### 3.6.2. Neurosurgical Cohort: Cranial vs. Spinal vs. Other Procedures

Neurosurgical interventions were stratified into three clinically relevant families:

- Cranial procedures: brain tumor resections.
- Spine surgery: discopathy, microdiscectomy, spinal cord injury surgery, vertebroplasty.
- Other neurosurgical procedures: thermolesion, hydrocephalus surgery, miscellaneous minor procedures.

This classification reflects distinct neuroinflammatory mechanisms and postoperative recovery profiles.

Pain intensity

- On day 1, cranial procedures exhibited higher mean NRS scores compared with spine surgery and other procedures (aligned with values shown in Table 3).
- On day 2, pain levels decreased more rapidly after spine operations, while cranial procedures showed a slower decline.

Delirium occurrence

- Severe delirium was most common in the spinal cord injury subgroup (5.83% on day 1), as presented in Table 5.
- Cranial procedures demonstrated a low but non-zero risk of delirium.
- Minor neurosurgical procedures showed minimal delirium incidence.

Interpretation

Stratification demonstrates that delirium risk varies across neurosurgical families even when pain levels are comparable. This supports the notion that delirium in neurosurgery is driven not only by pain intensity but also by neuroinflammatory burden, neurological status, and underlying disease.

## 4. Discussion

Pain and delirium remain clinically important postoperative complications, particularly among older adults undergoing major procedures [22]. In this retrospective analysis, we identified clear associations between postoperative pain intensity and delirium severity in patients following cardiac surgery, whereas no statistically significant relationships were observed in the neurosurgical group. These findings are consistent with prior research suggesting that pain and delirium can co-occur in vulnerable patient populations, although the mechanisms linking them are multifactorial and not yet fully understood [23,24,25].

Our observations should be interpreted in light of contemporary evidence on delirium pathogenesis. A comprehensive systematic review by Ormseth et al. [17], synthesizing 315 studies and more than 101,000 patients, identified 33 predisposing and 112 precipitating factors associated with delirium across healthcare settings. Among the precipitating variables, both surgical invasiveness and postoperative pain were explicitly recognized as contributors to delirium risk, though pain assessment methods varied widely, and standardized intensity scales were not consistently applied. These findings underscore that postoperative pain and type of procedure represent clinically meaningful and potentially modifiable factors interacting with patient vulnerability to influence delirium presentation. This framework supports the rationale behind our study, which focused specifically on pain intensity and procedure category in two high-risk surgical populations. At the same time, as emphasized by Ormseth et al., delirium emerges from a broad interplay of neurobiological and clinical factors, cautioning against overinterpretation of individual precipitants examined in isolation, especially within retrospective datasets [17].

In our cohort, cardiac surgery patients reported significantly higher pain intensity on postoperative day 1 compared with neurosurgical patients, aligning with previous reports in cardiac populations, despite variability in absolute pain scores between studies [26]. We also found a moderate positive correlation between NRS pain scores and CAM-ICU-7 delirium severity in the cardiac group on both postoperative days. This pattern is consistent with earlier studies showing that greater symptom burden—including pain—may accompany more severe delirium manifestations in postoperative patients [18,27]. However, due to the retrospective and observational design, these associations cannot be interpreted as causal.

A key challenge in interpreting the relationship between pain and delirium is the influence of perioperative pharmacologic management. Although both centers implemented standardized multimodal analgesia and sedation protocols, dosing, timing, and cumulative exposure to opioids, benzodiazepines, propofol, dexmedetomidine, anticholinergics, and antipsychotics were not consistently captured in medical records. This lack of detailed medication data precluded statistical adjustment for opioid burden (MME/day), benzodiazepine equivalents, sedative infusions, or anticholinergic load—factors well-established as influencing both pain perception and delirium risk. Similar limitations have been highlighted in other analyses exploring postoperative neurocognitive outcomes, where incomplete pharmacologic data introduce residual confounding and treatment-related bias [28,29,30,31]. Therefore, our findings should be interpreted cautiously and described as associations only.

An interesting observation in our dataset was that minimally invasive cardiac procedures (miniMVpl) were associated with lower mean NRS scores, while still presenting cases of mild–moderate and severe delirium. Conversely, more invasive procedures such as AVR, Bentall, and CABG—typically associated with higher postoperative pain—did not uniformly demonstrate elevated delirium rates. Comparable inconsistencies have been reported in previous cardiac surgery studies, where delirium risk appears to be influenced by a combination of age, comorbidities, perioperative inflammation, surgical complexity, and pharmacologic exposure rather than by pain intensity alone [30,31]. In our analysis, these findings should not be interpreted as evidence for or against a causal role of pain in specific cardiac subgroups, but rather as reflections of unmeasured interacting factors not fully accessible within this dataset.

In the neurosurgical group, the absence of a statistically significant association between pain intensity and delirium severity is consistent with the notion that pathophysiologic mechanisms of postoperative delirium in neurosurgical patients are often dominated by neuroinflammation, the underlying neurological condition, or perioperative cerebral stress, rather than by pain levels alone [16]. Spinal cord injury procedures were associated with the highest delirium incidence in our cohort, echoing observations in neurotrauma populations, which demonstrate heightened susceptibility to acute neurocognitive disturbances [1,13]. However, given the wide heterogeneity of neurosurgical diagnoses and operative techniques, these findings should be interpreted with caution.

Taken together, our results expand the limited literature comparing pain–delirium interactions across cardiac and neurosurgical surgical cohorts. While several significant associations were identified—primarily among cardiac surgery patients—the absence of comprehensive medication data and other potentially relevant confounders prevents any causal inference. Instead, our findings highlight the importance of prospective research incorporating standardized perioperative data collection, including detailed analgesic and sedative exposure, depth of sedation, inflammatory biomarkers, and longitudinal neurocognitive outcomes.

Despite the inherent limitations of retrospective observational research, this study provides meaningful insight into postoperative symptom patterns in two high-risk surgical groups. A better understanding of these relationships may support the development of more individualized postoperative monitoring strategies and inform the design of future prospective studies exploring the complex interactions among pain, neuroinflammation, perioperative care, and delirium.

An important methodological implication of our findings concerns the strong dependence of postoperative pain and delirium trajectories on exposure to analgesics, sedatives, anticholinergics, antipsychotics, and anesthetic techniques. Because individual-level pharmacologic data were unavailable, our analyses could not account for opioid burden (MME/day), benzodiazepine equivalents, propofol or dexmedetomidine exposure, or anticholinergic load—factors known to simultaneously influence both NRS pain scores and delirium severity. As a result, the observed associations between pain intensity and delirium—particularly in cardiac surgery patients—should be interpreted strictly as descriptive. They may reflect unmeasured treatment-related confounding rather than a direct clinical relationship. The absence of sensitivity analyses controlling for medication exposure further limits the extent to which causal inferences or mechanistic interpretations can be supported. Future research incorporating standardized perioperative pharmacologic documentation and formal confounder-adjusted modelling will be necessary to more reliably determine whether pain contributes to delirium risk beyond the effects of sedative and analgesic regimens.

### Strengths and Limitations

This study has several strengths. First, it is one of the few analyses to directly compare the courses of postoperative pain and delirium in two distinct groups of high-risk surgical patients—cardiac and neurosurgical—using standardized, validated instruments (NRS, CAM-ICU, CAM-ICU-7). Second, the dataset is characterized by exceptional completeness: all scheduled NRS assessments and all CAM-ICU/CAM-ICU-7 assessments were available, eliminating the risk of attrition bias and enabling robust nonparametric, logistic, and mixed-effects modeling. Third, the inclusion of repeated pain measurements at seven time points per day allows for detailed symptom dynamics rarely captured in retrospective analyses. Finally, the study contributes new comparative knowledge by examining differences between pain and delirium across surgical specialties with distinct neuroinflammatory and physiological profiles. Despite these strengths, several limitations should be noted. The most important limitation is the lack of individual-level data on perioperative and postoperative pharmacological exposure. Detailed information regarding opioid dosage (MME/day), benzodiazepine equivalents, propofol or dexmedetomidine infusions, anticholinergic load, and antipsychotic use was not consistently documented in the medical records. It, therefore, could not be included in the statistical models. Because these medications are known determinants of both pain intensity and the risk of delirium, the associations described in this study are highly sensitive to unmeasured confounders. Consequently, the observed associations between NRS scores and delirium severity should be interpreted strictly as descriptive, not causal, and may partially reflect treatment bias (indication confounder). Furthermore, although standardized institutional protocols for analgesia and sedation were described, the lack of quantitative data regarding dosage prevented sensitivity analyses or pharmacologically adjusted models. This limitation limits the comparability of our findings with the existing literature and limits the ability to draw more robust mechanistic conclusions. Finally, the retrospective design inherently limits temporal inferences, and unmeasured clinical variables such as preoperative cognitive status, frailty, sleep disturbances, or depth of anesthesia could not be fully controlled.

## 5. Conclusions

In this retrospective analysis, we identified significant associations between postoperative pain intensity and delirium severity among patients undergoing cardiac surgery, whereas no comparable relationship was observed in the neurosurgical cohort. These findings suggest that, in cardiac patients, higher pain levels and more severe delirium may co-occur; however, given the methodological constraints of the dataset, these observations should not be interpreted as evidence of a causal link.

A central limitation of this study is the absence of detailed, individual-level perioperative pharmacotherapy data—including opioid dosing, benzodiazepine exposure, sedative infusions, anticholinergic burden, and antipsychotic use. Because these medications influence both pain trajectories and delirium risk, the observed associations are highly susceptible to treatment-related confounding. As a result, all reported relationships must be regarded as descriptive and exploratory rather than inferential.

Despite these limitations, the study highlights the importance of consistent postoperative monitoring of both pain and cognitive status during the early postoperative period, particularly within the first 48 h after major cardiac or neurosurgical procedures. Early detection of abnormalities may support timely clinical interventions and improve patient safety.

The findings further underscore the need for prospective studies with standardized and comprehensive data collection, including detailed pharmacologic exposure, perioperative variables, inflammatory biomarkers, neurocognitive assessments, and validated measures of pain and delirium over time. Such rigorously designed research is essential to clarify whether postoperative pain contributes independently to delirium risk, or whether the relationship is primarily shaped by pharmacologic, neuroinflammatory, or patient-specific vulnerability factors.

Given the limitations related to unmeasured confounding, the associations described in this study should be considered hypothesis-generating. Future high-quality prospective cohorts or randomized trials are required to evaluate the complex interplay between pain, analgesic and sedative regimens, perioperative stress, neuroinflammatory mechanisms, and postoperative delirium.

## Figures and Tables

**Table 1:** Characteristics of grouping variables.

Characteristics of Grouping Variables							
Type	Category	Cardiac Surgery Patients		Neurosurgery Patients		X^2^	*p*
		n\202	%	n\206	%		
Gender	Female	90\202	44.55%	91\206	44.17%	0.01	0.93
	Male	112\202	55.45%	115\2026	55.83%		
Age group (year)	<30	17\202	8.42%	18\206	8.74%	26.57	0.00001
	31–45	4\202	19.80%	47\206	22.82%		
	46–60	74\202	36.63%	112\206	54.37%		
	>60	71\202	35.15%	29\206	14.08%		
ICU stay (hours)	0–24	69\202	34.16%	76\206	36.89%	3.03	0.21
	25–48	103\202	50.99%	89\206	43.20%		
	above 48	30\202	14.85%	41\206	19.90%		
Surgical procedures							
Patient Type	Type of treatment					n\202	%
Cardiac surgery patients	AVR (aortic valve replacement)					16\202	7.92%
	Bentall					13\202	6.44%
	miniMVpl (mini mitral valve plasty)					159\202	78.71%
	CABG (coronary artery bypass grafting)					14\202	6.93%
Neurosurgery patients	Type of treatment					n\206	%
	Discopathy					19\206	9.22%
	Brain tumor					26\206	12.62%
	Other					82\206	39.81%
	Micro discectomy of the lumbar spine					19\206	9.22%
	Thermolesion					25\206	12.14%
	Spinal cord injury					14\206	6.80%
	Vertebroplasty					12\206	5.83%
	Hydrocephalus					9\206	4.37%

**Table 2:** Level of pain in cardiac and neurosurgical patients.

NRS	Cardiac Surgery Patients					Neurosurgery Patients					*t*	*p*
	n	$\bar{x}$	Min	Max	SD	n	$\bar{x}$	Min	Max	SD		
Day 1st	202	5.32	2	8	1.25	206	4.74	1	8	2.12	−3.34	<0.000001
Day 2nd	202	4.52	2	8	1.14	206	4.62	2	8	1.4	0.78	0.43
t	6.74					0.71						
*p*	<0.000001					0.47						

NRS—Numerical Rating Scale; *p*—significance level; *t*—Student’s test; n—n-size; Min—minimum; Max—maximum; SD—standard deviation; $\bar{x}$—weighted average.

**Table 3:** NRS Pain Scale Level for Types of Procedures for Cardiac Surgery Patients.

NRS				Cardiac Surgery Patients														F		*p*
				AVR				Bentall					miniMVpl			CABG				
				$\bar{x}$		SD		$\bar{x}$		SD			$\bar{x}$		SD	$\bar{x}$	SD			
Day 1st				6.73		0.92		6.49		1.01			5.29		1.02	2.98	0.34	44.05		<0.00001
Day 2nd				4.27		1.04		5.19		1.50			4.62		1.06	2.98	0.34	12.43		<0.00001
t				7.12				2.61					5.75			0.00				
*p*				<0.00001				<0.00001					<0.00001			1.00				
NRS	Neurosurgery patients																			
	Discopathy		Brain tumor		Other		Micro discectomy of the lumbar spine		Thermolesion			Spinal cord injury			Vertebroplasty		Hydrocephalus		F	*p*
	$\bar{x}$	SD	$\bar{x}$	SD	$\bar{x}$	SD	$\bar{x}$	SD	$\bar{x}$		SD	$\bar{x}$		SD	$\bar{x}$	SD	$\bar{x}$	SD		
Day 1	5.19	1.12	6.42	1.76	5.58	1.26	2.20	0.15	0.99		0.41	6.16		1.47	4.88	1.07	4.75	1.32	62.58	<0.00001
Day 2	4.78	1.48	4.32	1.37	4.71	1.47	5.17	1.58	4.42		1.32	4.47		0.92	4.44	1.00	4.14	1.64	0.94	0.77
*t*	0.95		4.8		4.05		8.15		12.33			3.55			1.08		0.66			
*p*	0.34		0.00001		0.00004		<0.000001		<0.00001			0.001			0.31		0.4			

NRS—Numerical Rating Scale; *t*—Student test; *p*—significance level; SD—standard deviation; $\bar{x}$—weighted average; F—ANOVA analysis test; AVR—aortic valve replacement; CABG—coronary artery bypass grafting; miniMVpl—mini mitral valve plasty.

**Table 4:** Comparison of delirium levels in patient groups.

Delirium		Cardiac Surgery Patients									Neurosurgery Patients						F	*p*	
		n	$\bar{x}$		Min		Max		SD		n	$\bar{x}$	Min		Max	SD			
POD 1		202	1.62		0		7		2.35		206	1.07	0		7	1.59	7.86	0.005	
POD 2		202	1.15		0		7		1.62		206	0.52	0		7	0.74	25.63	0.000001	
Occurrence of different types of delirium in the study groups																			
Delirium	Cardiac surgery patients										Neurosurgery patients							X^2^	*p*
	No delirium			Mild-moderate delirium				Severe delirium			No delirium			Mild-moderate delirium		Severe delirium			
	n	%		n		%		n		%	n	%		n	%	n	%		
POD 1	154	76.24%		17		8.42%		31		15%	192	93.20%		1	0.49%	13	6.31%	28.19	<0.000001
POD 2	174	86.14%		18		8.91%		10		5%	204	99.03%		1	0.49%	1	0.49%	28.38	<0.000001

*p*—materiality level; SD—standard deviation; $\bar{x}$—weighted average; F—ANOVA analysis test; n—size; Min—minimum; Max—maximum; χ^2^—chi-square test; n—size; %—Percent; POD 1—postoperative day 1st; POD 2—postoperative day 2nd.

**Table 5:** Prevalence of delirium in cardiac and neurosurgical groups.

Cardiac Surgery Patients									χ^2^	*p*
Delirium	No Delirium		Mild-Moderate Delirium			Severe Delirium				
	n\202	%	n\202	%		n\202		%		
1st day										
AVR	6\202	3%	2\202	0.99%		8\202		3.96%	42.01	<0.000001
Bentall	3\202	1%	2\202	0.99%		8\202		3.96%		
miniMVpl	131\202	64.85%	13\202	6.44%		15\202		7.43%		
CABG	14\202	6.93%	0\202	0%		0\202		0%		
2nd day										
AVR	13\202	6%	3\202	1.49%		0\202		0%	11.32	0.07
Bentall	11\202	5%	0\202	0.00%		2\202		1%		
miniMVpl	136\202	67.33%	15\202	7.43%		8\202		4%		
CABG	14\202	7%	0\202	0%		0\202		0%		
Neurosurgery patients									χ^2^	*p*
Delirium		No delirium		Mild-moderate delirium			Severe delirium			
		n\206	%	n\206	%		n\206	%		
1st day										
Discopathy		18\206	8.74%	0\206	0%		0\206	0.00%	87.42	<0.000001
Brain tumor		26\206	12.62%	0\206	0%		0\206	0%		
Other		82\206	39.81%	0\206	0%		0\206	0%		
Micro discectomy of the lumbar spine		18\206	8.74%	0\206	0%		1\206	0.49%		
Thermolesion		25\206	12.14%	0\206	0%		0\206	0%		
Spinal cord injury		1\206	0.49%	1\206	0.49%		12\206	5.83%		
Vertebroplasty		12\206	5.83%	0\206	0%		0\206	0%		
Hydrocephalus		9\206	4.37%	0\206	0%		0\206	0%		
2nd day										
Discopathy		19\206	9.22%	0\206	0%		0\206	0%	11.05	0.68
Brain tumor		28\206	13.59%	0\206	0%		0\206	0%		
Other		82\206	39.81%	0\206	0%		0\206	0%		
Micro discectomy of the lumbar spine		19\206	9.22%	0\206	0%		0\206	0%		
Thermolesion		25\206	12.14%	0\206	0%		0\206	0%		
Spinal cord injury		12\206	5.83%	1\206	0.49%		1\206	0.49%		
Vertebroplasty		12\206	5.83%	0\206	0%		0\206	0%		
Hydrocephalus		9\206	4.37%	0\206	0%		0\206	0%		

*p*—significance level; n—size; χ^2^—chi-square test; AVR—aortic valve replacement; CABG—coronary artery bypass grafting; miniMVpl—mini mitral valve plasty.

**Table 6:** Spearman correlation between pain and CAM-ICU-7.

Pairwise Comparison	ρ	95% CI	*p* (Holm)
NRS 1st day vs. CAM-ICU-7 POD 1	0.23	0.14–0.32	0.000007
NRS 2nd day vs. CAM-ICU-7 POD 2	0.04	−0.06–0.13	0.4546

ρ—Spearman rank correlation coefficient; CI—confidence interval; *p* (Holm)—Holm-adjusted *p*-value.

**Table 7:** Mann–Whitney U test: pain vs. delirium presence.

Variable	Median (No Delirium)	Median (Delirium)	r	*p* (Holm)
NRS 1st day	5.29	6.00	0.25	0.000002
NRS 2nd day	4.57	4.71	0.08	0.1747

r—effect size; *p* (Holm)—Holm-adjusted *p*-value.

**Table 8:** Kruskal–Wallis analysis of pain and delirium on the 1st day.

Delirium Category	n\408 (%)	Median
0–2 points (no delirium)	346\408 (84.8%)	5.29
3–5 points (mild/moderate)	18\408 (4.4%)	6.14
6–7 points (severe)	44\408 (10.8%)	6.14

n—number of patients included in each delirium severity category.

**Table 9:** Kruskal–Wallis analysis of pain and delirium on the 2nd day.

Delirium Category	n\408 (%)	Median
0–2 points (no delirium)	378/408 (92.6%)	4.57
3–5 points (mild/moderate)	19/408 (4.7%)	5.14
6–7 points (severe)	11/408 (2.7%)	5.57

n—number of patients included in each delirium severity category.

**Table 10:** Dunn post hoc for NRS 1st day and delirium.

Delirium Category (Group A)	Delirium Category (Group B)	Mean Rank A	Mean Rank B	Δ Ranks	z	*p* (Holm)
No delirium	Mild/moderate delirium	188.99	286.00	97.01	3.40	0.0013
No delirium	Severe delirium	188.88	293.13	104.14	5.52	<0.000001
Mild/moderate delirium	Severe delirium	286.00	293.13	7.13	0.22	0.8299

Δ ranks—absolute difference in mean ranks; z—standardized Dunn statistic; *p* (Holm)—Holm-adjusted *p*-value.

**Table 11:** Dunn post hoc for NRS 2nd day and delirium.

Delirium Category (Group A)	Delirium Category (Group B)	Mean Rank A	Mean Rank B	Δ Ranks	z	*p* (Holm)
No delirium	Mild/moderate delirium	199.52	240.45	40.93	1.48	0.1399
No delirium	Severe delirium	199.52	313.55	114.03	3.16	0.0047
Mild/moderate delirium	Severe delirium	240.45	313.55	73.10	1.64	0.2036

Δ ranks—absolute difference in mean ranks; z—standardized Dunn statistic; *p* (Holm)—Holm-adjusted *p*-value.

**Table 12:** Mixed-effect for repeated pain measurements.

Variable	β	95% CI	*p*
Intercept	4.58	4.53–4.81	<0.001
Time (linear trend)	0.07	0.03–0.11	<0.001
Delirium	1.23	0.99–1.48	<0.001

β—regression coefficient; CI—95% confidence interval; *p*—*p*-value.

**Table 13:** Multivariate analysis.

Variable	OR	95% CI	*p*
Age (year)	1.00	0.98–1.02	0.7847
Sex (male vs. female)	1.12	0.66–1.90	0.6662
Procedure (Neurosurgery vs. Cardiosurgery)	0.15	0.08–0.29	<0.001
Number of comorbidities	0.81	0.59–1.12	0.2019
ICU stay (hour)	1.01	1.00–1.02	0.0981

OR—odds ratio; CI—confidence interval; *p*—*p*-value.

**Table 14:** Ordinal logistic regression for CAM-ICU-7 delirium severity.

Variable	OR	95% CI	*p*
NRS 1st day	2.24	1.70–2.94	<0.001
Age (year)	0.99	0.97–1.01	0.488
Sex (male vs. female)	0.74	0.41–1.34	0.318
Procedure type	0.19	0.09–0.40	<0.001

OR—odds ratio; CI—confidence interval; *p*—*p*-value.

## Data Availability

The original contributions presented in this study are included in the article. Further inquiries can be directed to the corresponding author.

## Abbreviations

The following abbreviations are used in this manuscript:

ICU	Intensive Care Unit
NRS	Numerical Rating Scale
CABG	Coronary artery bypass grafting
miniMVpl	Mini mitral valve plasty
AVR	Aortic valve replacement
SD	Standard deviation
CAM-ICU	Confusion Assessment Method for the Intensive Care Unit
POD 1	Postoperative delirium day 1
POD 2	Postoperative delirium day 2

## References

[1] Brown C.H., LaFlam A., Max L., Wyrobek J., Neufeld K.J., Kebaish K.M., Cohen D.B., Walston J.D., Hogue C.W., Riley L.H. (2016). Delirium After Spine Surgery in Older Adults: Incidence, Risk Factors, and Outcomes. J. Am. Geriatr. Soc..

[2] Sato T., Hatakeyama S., Okamoto T., Yamamoto H., Hosogoe S., Tobisawa Y., Yoneyama T., Hashiba E., Yoneyama T., Hashimoto Y. (2016). Slow Gait Speed and Rapid Renal Function Decline Are Risk Factors for Postoperative Delirium after Urological Surgery. PLoS ONE.

[3] Jin Z., Hu J., Ma D. (2020). Postoperative delirium: Perioperative assessment, risk reduction, and management. Br. J. Anaesth..

[4] Cierzniakowska K., Popow A., Kozłowska E., Magdzińska M., Samodulska A., Jabłońska R., Szewczyk M.T. (2023). Delirium syndrome in the postoperative period. Pielęgniarstwo Chir. I Angiol./Surg. Vasc. Nurs..

[5] Winter A., Steurer M.P., Dullenkopf A. (2015). Postoperative delirium assessed by post anesthesia care unit staff utilizing the Nursing Delirium Screening Scale: A prospective observational study of 1000 patients in a single Swiss institution. BMC Anesthesiol..

[6] Ha A., Krasnow R.E., Mossanen M., Nagle R., Hshieh T.T., Rudolph J.L., Chang S.L. (2018). A contemporary population-based analysis of the incidence, cost, and outcomes of postoperative delirium following major urologic cancer surgeries. Urol. Oncol..

[7] Drews T., Franck M., Radtke F.M., Weiss B., Krampe H., Brockhaus W.R., Winterer G., Spies C.D. (2015). Postoperative delirium is an independent risk factor for posttraumatic stress disorder in the elderly patient: A prospective observational study. Eur. J. Anaesthesiol..

[8] Daiello L.A., Racine A.M., Gou R.Y., Marcantonio E.R., Xie Z., Kunze L.J., Vlassakov K.V., Inouye S.K., Jones R.N., SAGES Study Group (2019). Postoperative delirium and postoperative cognitive dysfunction: Overlap and divergence. Anesthesiology.

[9] Inouye S.K., Marcantonio E.R., Kosar C.M., Tommet D., Schmitt E.M., Travison T.G., Saczynski J.S., Ngo L.H., Alsop D.C., Jones R.N. (2016). The short-term and long-term relationship between delirium and cognitive trajectory in older surgical patients. Alzheimers Dement..

[10] Ansaloni L., Catena F., Chattat R., Fortuna D., Franceschi C., Mascitti P., Melotti R.M. (2010). Risk factors and incidence of postoperative delirium in elderly patients after elective and emergency surgery. Br. J. Surg..

[11] Camous J., Decrombecque T., Louvain-Quintard V., Doubine S., Dartevelle P., Stéphan F. (2014). Outcomes of patients with antiphospholipid syndrome after pulmonary endarterectomy. Eur. J. Cardiothorac. Surg..

[12] Chaiwat O., Chanidnuan M., Pancharoen W., Vijitmala K., Danpornprasert P., Toadithep P., Thanakiattiwibun C. (2019). Postoperative delirium in critically ill surgical patients: Incidence, risk factors, and predictive scores. BMC Anesthesiol..

[13] Sampson E.L., West E., Fischer T. (2020). Pain and delirium: Mechanisms, assessment, and management. Eur. Geriatr. Med..

[14] Wilson J.E., Mart M.F., Cunningham C., Shehabi Y., Girard T.D., MacLullich A.M., Slooter A.J., Ely E.W. (2020). Delirium. Nat. Rev. Dis. Primers.

[15] American Psychiatric Association (2013). Diagnostic and Statistical Manual of Mental Disorders.

[16] Kappen P.R., Kakar E., Dirven C.M.F., van der Jagt M., Klimek M., Osse R.J., Vincent A.P.J.E. (2022). Delirium in neurosurgery: A systematic review and meta-analysis. Neurosurg. Rev..

[17] Ormseth C.H., LaHue S.C., Oldham M.A., Josephson S.A., Whitaker E., Douglas V.C. (2023). Predisposing and Precipitating Factors Associated With Delirium: A Systematic Review. JAMA Netw. Open.

[18] White N., Bazo-Alvarez J.C., Koopmans M., West E., Sampson E.L. (2024). Understanding the association between pain and delirium in older hospital inpatients: Systematic review and meta-analysis. Age Ageing.

[19] Weisse A.B. (2011). Cardiac surgery: A century of progress. Tex. Heart Inst. J..

[20] Gilard V., Derrey S., Marret S., Bekri S., Tebani A. (2021). Precision Neurosurgery: A Path Forward. J. Pers. Med..

[21] Khan B.A., Perkins A.J., Gao S., Hui S.L., Campbell N.L., Farber M.O., Chlan L.L., Boustani M.A. (2017). The Confusion Assessment Method for the ICU-7 Delirium Severity Scale: A Novel Delirium Severity Instrument for Use in the ICU. Crit. Care Med..

[22] Zhang B., Zhang Z. (2016). Mediation analysis to unravel mechanisms underlying association between platelet transfusion and postoperative delirium. Crit. Care.

[23] Zhang Z. (2023). Editorial: Postoperative care: From pain management to delirium. Front. Med..

[24] Fong T.G., Vasunilashorn S.M., Ngo L., Libermann T.A., Dillon S.T., Schmitt E.M., Pascual-Leone A., Arnold S.E., Jones R.N., Marcantonio E.R. (2020). Association of Plasma Neurofilament Light with Postoperative Delirium. Ann. Neurol..

[25] Bonnet F., Marret E. (2007). Postoperative pain management and outcome after surgery. Best. Pract. Res. Clin. Anaesthesiol..

[26] Micah S., Barolia R., Parpio Y., Kumar S., Sharif H. (2019). Factors Associated with Postoperative Pain among Patients after Cardiac Surgery in the Tertiary Care Teaching Hospital of Karachi, Pakistan. Pain. Res. Treat..

[27] Baltazar L.F.S.R., Reis G.B.B., Paiva A.M.d., Silva P.G.M.d., Souza A.H.d., Gardenghi G. (2023). Delirium and pain in patients in the immediate postoperative period of cardiac surgery: Prevalence and associated risk factors/Delirium e dor em pacientes no pós-operatório imediato de cirurgia cardíaca: Prevalência e fatores de risco associados. BrJP.

[28] Marcantonio E.R. (2012). Postoperative delirium: A 76-year-old woman with delirium following surgery. JAMA.

[29] Bilotta F., Lauretta M.P., Borozdina A., Mizikov V.M., Rosa G. (2013). Postoperative delirium: Risk factors, diagnosis and perioperative care. Minerva Anestesiol..

[30] Russell M.D., Pinkerton C., Sherman K.A., Ebert T.J., Pagel P.S. (2020). Predisposing and Precipitating Factors Associated With Postoperative Delirium in Patients Undergoing Cardiac Surgery at a Veterans Affairs Medical Center: A Pilot Retrospective Analysis. J. Cardiothorac. Vasc. Anesth..

[31] Gosselt A.N., Slooter A.J., Boere P.R., Zaal I.J. (2015). Risk factors for delirium after on-pump cardiac surgery: A systematic review. Crit. Care.

